# *Candida parapsilosis* and echinocandins: should the clinical laboratory drop anidulafungin Etest from the susceptibility testing panel?

**DOI:** 10.1128/aac.00241-25

**Published:** 2025-07-01

**Authors:** Roya Vahedi-Shahandashti, Eleonora Moroder, Cornelia Lass-Flörl

**Affiliations:** 1 Institute of Hygiene and Medical Microbiology, Medical University of Innsbruck 172142, Innsbruck, Austria; University of Iowa, Iowa City, Iowa, USA

**Keywords:** *Candida parapsilosis*, echinocandins, Etest, anidulafungin, echinocandins resistance, candidiasis, *fks *gene

## Abstract

Antifungal susceptibility testing (AFST) is essential for clinical decisions to effectively select optimal therapy against fungal infections. Reference standard broth microdilution methods provide accurate results; however, they are more challenging and time-consuming compared to conventional susceptibility testing methods such as gradient diffusion strips (Etest). This study assessed the Clinical and Laboratory Standards Institute (CLSI) yeast protocol and Etest for determining the echinocandin susceptibility profile (anidulafungin, caspofungin, micafungin, and rezafungin) in *Candida parapsilosis* isolates (*n* = 60). A full agreement (100%) was observed between Etest and CLSI for caspofungin, micafungin, and rezafungin, but surprisingly, the agreement for anidulafungin was only 43.3%. Notably, 56.6% of isolates were anidulafungin resistant by Etest, whereas CLSI detected no resistance. Analysis of *fks*1 gene hotspots (HS1 and HS2) revealed no mutations, proving that anidulafungin susceptibility results are method-dependent rather than a consequence of actual resistance. Our findings suggest that Etest may overestimate anidulafungin resistance in *C. parapsilosis*, which can lead to false resistance categorization. The low agreement between the two methods, Etest and CLSI, highlights the need to address these issues through improved quality control of the anidulafungin Etest or methodological adjustments. Given that anidulafungin is considered the leading drug in AFST for *C. parapsilosis*, caution is recommended when interpreting Etest results without confirmation via broth microdilution. Since these discrepancies in categorization occur for a specific species and a particular antifungal, anidulafungin, within the echinocandin class, rather than for all agents in that class (with anidulafungin also serving as the reference agent in AFST), this may suggest that, in the case of *C. parapsilosis*, either an alternative method should be used for anidulafungin susceptibility testing, or other echinocandins should be considered as reference agents in clinical settings.

## INTRODUCTION

Candidemia remains a prevalent and life-threatening infection, leading to considerable morbidity and mortality, particularly in critically ill patients (1, 2). Invasive candidiasis affects over 250,000 people worldwide annually, with a mortality rate exceeding 50,000 (3, 4). While *Candida albicans* was historically considered the predominant species in candidiasis cases (1), there has been a notable transition from *C. albicans*-associated infections to those caused by non-*albicans Candida* species, such as *Candida glabrata*, *Candida parapsilosis*, *Candida krusei*, and *Candida tropicalis* (5, 6). *C. parapsilosis* is among the second to fourth most commonly identified species causing candidemia, with its prevalence varying depending on patient age and geographic location (7). Infections caused by *C. parapsilosis* are primarily observed in individuals admitted to intensive care units, affecting specific populations such as low-birth-weight neonates, transplant recipients, critical care patients, and individuals undergoing parenteral nutrition (8). *C. parapsilosis* stands out among *Candida* species due to its high transmissibility and unique colonization patterns, contributing to its persistence and prevalence in healthcare settings (1, 8). Additionally, the growing emergence of fluconazole-resistant *C. parapsilosis* in numerous countries is alarming due to its association with high mortality rates (9, 10).

Echinocandins are considered the first-line empirical therapy for suspected or confirmed disseminated candidiasis, followed by azoles and amphotericin B, depending on the *Candida* species and the patient’s prior exposure or intolerance to an antifungal agent (1, 11). Although *C. parapsilosis* usually shows higher echinocandins minimum inhibitory concentrations (MICs) than other *Candida* species (12), patients with systemic infections caused by *C. parapsilosis* generally respond well to echinocandin treatments (13). Echinocandins interfere with β-d-glucan synthase, which is encoded by two genes, *fks1* and *fks2*, showing excellent fungicidal activity against most *Candida* spp. (14). Echinocandin resistance in *Candida* species is primarily due to mutations in two highly conserved hot spots of the β-1,3-d-glucan synthase catalytic subunits, *fks*1 and *fks*2 (15). Besides hot spot regions, mutations outside the hotspot could also facilitate the development of resistance and tolerance to echinocandins (16). In addition, heteroresistance—a phenotypically unstable, low-frequency subpopulation of resistant cells—may preclude resistance and, in the case of *C. parapsilosis*, has been linked to prophylaxis failure and increasing the risk of breakthrough infections (17).

Echinocandin susceptibility testing is advisable for patients previously treated with echinocandins and those with *C. parapsilosis* infection, given the potential for breakthrough infections (18). As broth microdilution antifungal susceptibility testing (AFST) methods are labor-intensive and time-consuming, commercial agar-based AFST methods, such as gradient diffusion strips (e.g., Etest), were found to be a convenient technique in clinical practice, resulting in faster turnaround times and simplifying the inhibitory value evaluation method (19, 20). However, some discrepancies between the Etest and broth microdilution results were detected for echinocandins against some *Candida* species (21, 22), raising the question of which antifungal and which susceptibility testing method to use, especially when treating *C. parapsilosis*. To focus only on *C. parapsilosis* species and also to evaluate the suitability of echinocandins Etest results in the routine clinical setting, the susceptibility profile of echinocandins (anidulafungin, caspofungin, micafungin, and rezafungin) against *C. parapsilosis* (*n* = 60) was performed and compared to those MICs obtained by the Clinical and Laboratory Standards Institute (CLSI) method. In addition, the potential impact of mutations in the hot spot regions of the *fks*1 gene on susceptibility results was also analyzed.

## RESULTS

### Method-dependent shift in anidulafungin MIC distribution

**Fig 1:**
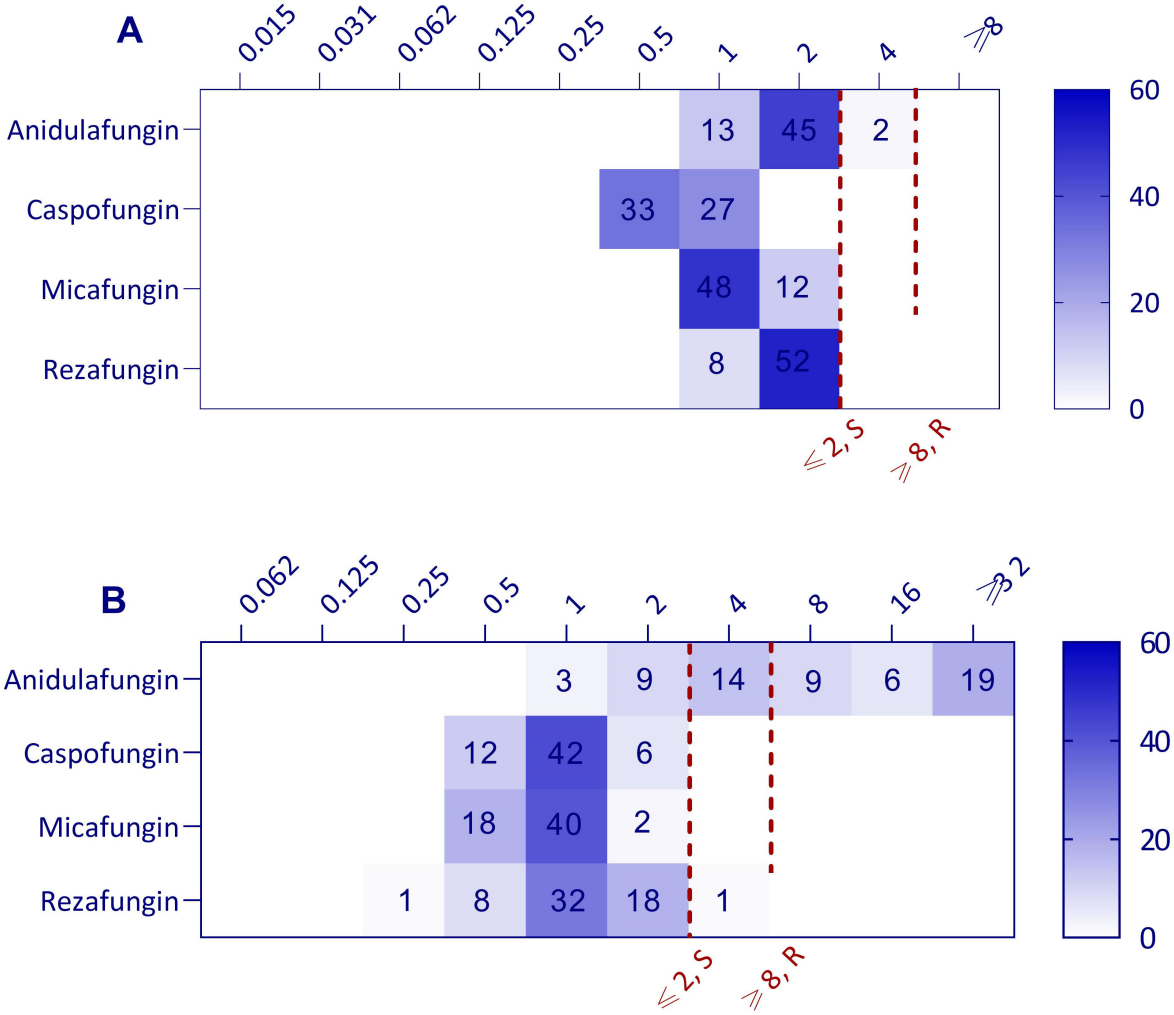
Echinocandin susceptibility profile of *Candida parapsilosis*. Heat map illustrating the distribution of minimum inhibitory concentrations (MICs) (µg/mL) of tested echinocandins, including anidulafungin, caspofungin, micafungin, and rezafungin against *C. parapsilosis* (*n* = 60), with data represented by the absolute number of isolates rather than percentages, according to the Clinical and Laboratory Standards Institute (CLSI) (**A**) and Etest (**B**) methods. According to the CLSI breakpoints (M27M44S-Ed3), all echinocandins are categorized as follows: ≤2; susceptible (S), =4; intermediate (I), and ≥8; and resistant (R). An exception is rezafungin, for which the only available categorization is ≤2; susceptible (S). Etest MICs were rounded up to the next higher log2 dilution to facilitate comparison with broth microdilution results.

The MIC distributions of four echinocandins against 60 clinical isolates of *C. parapsilosis*, determined by the CLSI and Etest methods, revealed a significant method-dependent shift for anidulafungin, showing a higher MIC range by Etest (1 to >32 µg/mL) compared to CLSI (1–4 µg/mL), while other tested echinocandins did not show a shift (Fig. 1). Of the four echinocandins tested by Etest, anidulafungin exhibited the highest MIC values, ranging from 1 to >32 µg/mL, followed by rezafungin with a MIC range of 0.25–4 µg/mL. Caspofungin and micafungin showed better activity, with MIC values ranging between 0.5 and 2 µg/mL (Fig. 1; Table 1). According to the CLSI method, anidulafungin displayed the highest MIC range (1–4 µg/mL), while caspofungin (0.5–1 µg/mL), micafungin, and rezafungin (both ranging from 1 to 2 µg/mL) demonstrated lower MIC range as illustrated in Fig. 1 and Table 1. In addition to the 60 clinical isolates, three *C. parapsilosis fks1*^R658G^ mutant isolates were also tested for all four echinocandins using both the Etest and CLSI methods (Table 2). The MIC results for these mutant isolates are presented separately from the other isolates to minimize bias and ensure that the MIC range for each agent and its essential agreement (EA) remain neutral and are not influenced by the inclusion of mutant strains (Table 2). Mutant isolates were previously characterized and categorized as anidulafungin susceptible, micafungin resistant, and caspofungin intermediate according to CLSI (23, 24). The MIC of the mutant isolates in this study was determined using both the CLSI and Etest methods. According to CLSI, the isolates were categorized as anidulafungin-susceptible, micafungin-resistant, caspofungin-intermediate, and rezafungin-intermediate, aligning with previous findings (23, 24). In contrast, Etest classified the isolates as anidulafungin-resistant, micafungin-resistant, caspofungin-intermediate, and rezafungin-intermediate, highlighting a lack of agreement between the two methods only for anidulafungin.

**TABLE 1:** The susceptibility profiles of anidulafungin (AND), caspofungin (CS), micafungin (MYC), and rezafungin (RZF) against *Candida parapsilosis* isolates using the Clinical and Laboratory Standards Institute (CLSI) and Etest methodologies

Isolates (no.)	AFST method	Antifungal agents/MIC (µg/mL)			
		AND	CS	MYC	RZF
*C. parapsilosis* (R1)	CLSI	4	1	2	2
	Etest	>32	1	1	4
*C. parapsilosis* (R2)	CLSI	2	1	1	2
	Etest	4	0.5	0.5	1
*C. parapsilosis* (R3)	CLSI	2	0.5	1	2
	Etest	32	1	1	2
*C. parapsilosis* (R4)	CLSI	2	0.5	1	2
	Etest	>32	0.5	1	1
*C. parapsilosis* (R5)	CLSI	2	0.5	2	2
	Etest	16	1	1	2
*C. parapsilosis* (R6)	CLSI	2	0.5	2	2
	Etest	>32	1	1	2
*C. parapsilosis* (R7)	CLSI	1	1	1	2
	Etest	2	1	0.5	1
*C. parapsilosis* (R8)	CLSI	2	0.5	2	2
	Etest	>32	0.5	1	1
*C. parapsilosis* (R9)	CLSI	4	0.5	2	2
	Etest	>32	0.5	1	2
*C. parapsilosis* (R10)	CLSI	2	0.5	2	2
	Etest	>32	0.5	1	2
*C. parapsilosis* (R11)	CLSI	2	0.5	2	2
	Etest	>32	1	1	2
*C. parapsilosis* (R12)	CLSI	1	0.5	1	1
	Etest	2	1	0.5	0.5
*C. parapsilosis* (R13)	CLSI	2	0.5	1	2
	Etest	32	2	1	1
*C. parapsilosis* (R14)	CLSI	2	0.5	1	2
	Etest	16	1	1	2
*C. parapsilosis* (R15)	CLSI	2	0.5	1	2
	Etest	4	1	1	1
*C. parapsilosis* (R16)	CLSI	2	0.5	1	2
	Etest	16	1	1	2
*C. parapsilosis* (R17)	CLSI	2	0.5	1	2
	Etest	>32	1	1	1
*C. parapsilosis* (R18)	CLSI	2	0.5	1	2
	Etest	>32	1	1	2
*C. parapsilosis* (R19)	CLSI	2	0.5	1	2
	Etest	4	1	1	1
*C. parapsilosis* (R20)	CLSI	2	0.5	1	2
	Etest	>32	2	1	2
*C. parapsilosis* (R21)	CLSI	2	0.5	1	2
	Etest	8	1	1	1
*C. parapsilosis* (R22)	CLSI	2	0.5	1	2
	Etest	>32	0.5	1	1
*C. parapsilosis* (R23)	CLSI	2	0.5	1	2
	Etest	32	0.5	1	1
*C. parapsilosis* (R24)	CLSI	2	1	2	2
	Etest	>32	2	1	2
*C. parapsilosis* (I1)	CLSI	2	0.5	1	2
	Etest	2	1	0.5	1
*C. parapsilosis* (I2)	CLSI	2	1	1	2
	Etest	16	1	1	1
*C. parapsilosis* (I3)	CLSI	2	1	1	2
	Etest	8	1	1	1
*C. parapsilosis* (I4)	CLSI	2	0.5	1	2
	Etest	16	1	1	1
*C. parapsilosis* (I5)	CLSI	1	1	1	2
	Etest	4	1	1	0.5
*C. parapsilosis* (I6)	CLSI	2	0.5	1	2
	Etest	8	1	1	1
*C. parapsilosis* (I7)	CLSI	2	1	2	2
	Etest	8	0.5	0.5	1
*C. parapsilosis* (I8)	CLSI	2	1	2	2
	Etest	4	1	1	1
*C. parapsilosis* (I9)	CLSI	1	0.5	1	1
	Etest	4	1	1	1
*C. parapsilosis* (I10)	CLSI	2	0.5	1	2
	Etest	8	0.5	1	1
*C. parapsilosis* (I11)	CLSI	1	0.5	1	1
	Etest	4	1	0.5	1
*C. parapsilosis* (I12)	CLSI	1	0.5	1	2
	Etest	2	0.5	0.5	0.5
*C. parapsilosis* (I13)	CLSI	2	1	1	2
	Etest	8	2	1	2
*C. parapsilosis* (I14)	CLSI	1	0.5	1	2
	Etest	1	1	0.5	0.5
*C. parapsilosis* (I15)	CLSI	2	1	1	2
	Etest	8	1	1	1
*C. parapsilosis* (I16)	CLSI	2	1	2	2
	Etest	4	1	1	1
*C. parapsilosis* (I17)	CLSI	2	1	1	2
	Etest	4	1	1	2
*C. parapsilosis* (I18)	CLSI	2	1	1	2
	Etest	8	1	0.5	1
*C. parapsilosis* (I19)	CLSI	2	0.5	1	2
	Etest	16	1	1	1
*C. parapsilosis* (I20)	CLSI	2	1	1	1
	Etest	4	1	0.5	0.5
*C. parapsilosis* (I21)	CLSI	2	1	1	2
	Etest	>32	1	1	2
*C. parapsilosis* (I22)	CLSI	2	1	1	1
	Etest	2	1	0.5	0.5
*C. parapsilosis* (I23)	CLSI	1	1	1	2
	Etest	4	1	0.5	1
*C. parapsilosis* (I24)	CLSI	1	1	1	2
	Etest	4	1	1	1
*C. parapsilosis* (S1)	CLSI	2	0.5	1	2
	Etest	8	1	1	2
*C. parapsilosis* (S2)	CLSI	1	0.5	1	1
	Etest	2	0.5	0.5	0.25
*C. parapsilosis* (S3)	CLSI	1	0.5	1	1
	Etest	1	1	0.5	0.5
*C. parapsilosis* (S4)	CLSI	1	0.5	1	1
	Etest	1	0.5	0.5	1
*C. parapsilosis* (S5)	CLSI	2	1	1	2
	Etest	2	1	0.5	1
*C. parapsilosis* (S6)	CLSI	1	1	1	2
	Etest	4	1	1	0.5
*C. parapsilosis* (S7)	CLSI	2	1	1	2
	Etest	2	1	0.5	1
*C. parapsilosis* (S8)	CLSI	2	1	1	2
	Etest	4	2	0.5	1
*C. parapsilosis* (S9)	CLSI	2	1	1	2
	Etest	>32	1	2	2
*C. parapsilosis* (S10)	CLSI	2	1	1	2
	Etest	2	1	1	1
*C. parapsilosis* (N1)	CLSI	2	1	1	2
	Etest	32	1	1	2
*C. parapsilosis* (N2)	CLSI	2	1	2	2
	Etest	>32	2	2	2
GM^*a*^	CLSI	1.76	0.68	1.15	1.8
	Etest	8.28	0.93	0.83	1.12
MIC_90_^*b*^	CLSI	2	1	2	2
	Etest	>32	1	1	2
MIC range	CLSI	1–4	0.5–1	1–2	1–2
	Etest	1–>32	0.5–2	0.5–2	0.25–4
Categorization^*c*^^,*e*^	CLSI (S) (%)	96.6	100	100	100
	CLSI (I) (%)	3.3	–^*f*^	–	–
	CLSI (R) (%)	–	–	–	–
	Etest (S) (%)	20	100	100	98.3
	Etest (I) (%)	23.3	–	–	–
	Etest (R) (%)	56.6	–	–	–
EA^*d*^ of CLSI vs Etest^*e*^ (%)		43.3	100	100	100


^*a*^ Geometric mean (GM).

^*b*^ Minimum inhibitory concentrations 90 (MIC_90_) (MICs inhibiting ≥90% of tested isolates).

^*c*^ According to the CLSI breakpoints (M27M44S-Ed3); S, Susceptible; I, Intermediate; and R, Resistant.

^*d*^ Essential agreement (EA).

^*e*^ Those Etest MICs that were not in the range of microdilution, were rounded up to the next higher log_2_ dilution.

^*f*^ A dash indicates that no isolates fell into that specific category.

**TABLE 2:** The antifungal susceptibility profile of anidulafungin (AND), caspofungin (CS), micafungin (MYC), and rezafungin (RZF) against *Candida parapsilosis* isolates harboring the *fks1^R658G^* mutation, as determined by the Clinical and Laboratory Standards Institute (CLSI) and Etest methodologies

Isolates (no.)	AFST method	Antifungal agents/MIC (µg/mL)			
		AND	CS	MYC	RZF
*C. parapsilosis fks1^R658G^* (CP 207)	CLSI	2	4	>8	4
	Etest	>32	4	>32	4
*C. parapsilosis fks1^R658G^* (CP 120)	CLSI	2	4	>8	4
	Etest	>32	4	>32	4
*C. parapsilosis fks1^R658G^* (CP 142)	CLSI	2	4	>8	4
	Etest	>32	4	>32	4

**Fig 2:**
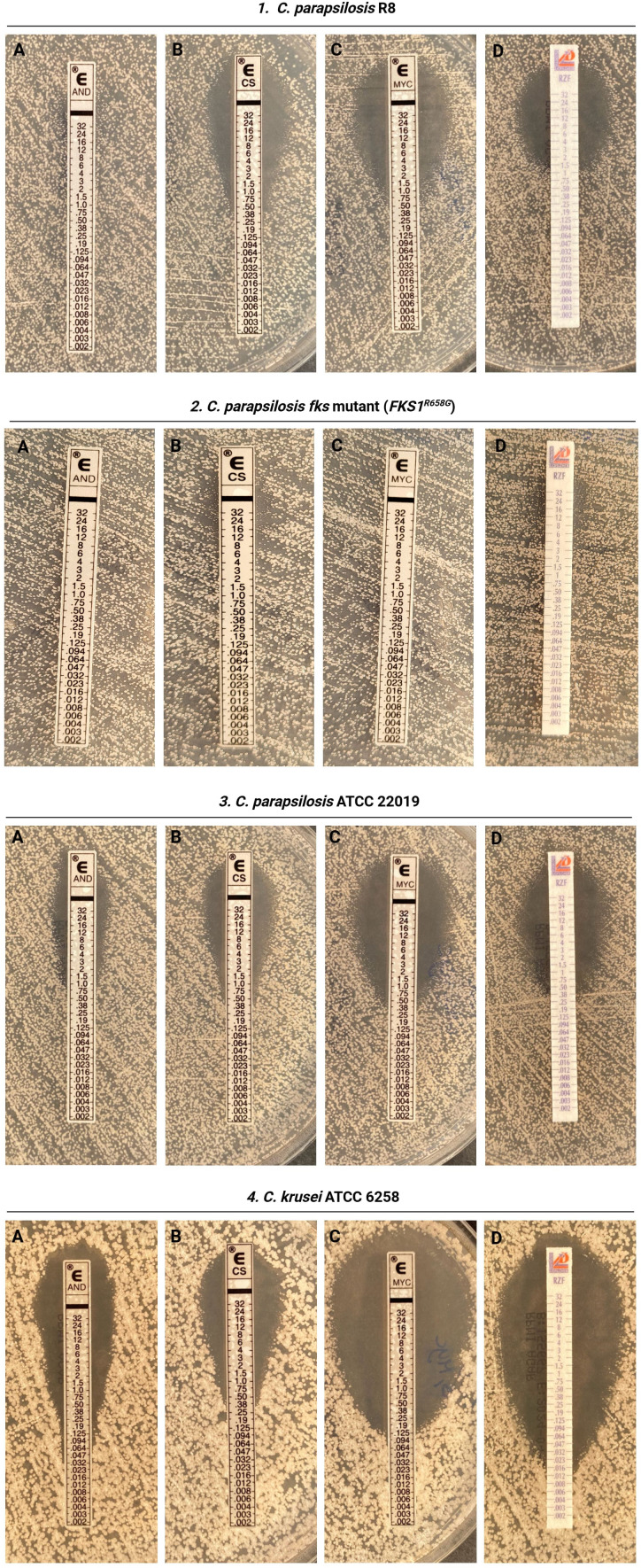
Inhibition pattern of a representative clinical *Candida parapsilosis* isolate (Isolate no. R8) (1), susceptible to anidulafungin (AND) (**A**) by CLSI but resistant by Etest , while susceptible to caspofungin (CS) (**B**), micafungin (MYC) (**C**), and rezafungin (RZF) (**D**) by both methods; (2) *C. parapsilosis* isolate *fks*1^R658G^ mutant (CP142), classified by CLSI as AND-susceptible, MYC-resistant, and RZF and CS-intermediate, but by Etest showing resistance to AND (**A**) and MYC (**C**), and intermediate to RZF (**D**), and CS (**B**); (3) *C. parapsilosis* ATCC 22019 and (4) *C. krusei* ATCC 6258 as quality control strains, both within the accepted CLSI quality control range.

Based on the breakpoints provided by CLSI, all isolates were categorized as susceptible to caspofungin and micafungin using both the CLSI and Etest methods. MICs obtained for rezafungin by CLSI categorized all isolates as susceptible, whereas by Etest, 98.3% were susceptible (Fig. 1; Table 1). The most notable discrepancy in susceptibility categorization was observed for anidulafungin. According to CLSI breakpoints, 96.6% of the isolates were classified as susceptible using the CLSI method, whereas Etest identified only 20% as susceptible (Fig. 1; Table 1). Additionally, CLSI did not categorize any of the isolates as anidulafungin resistant (3.3% intermediate), while Etest classified 56.6% of isolates as resistant (Fig. 1; Table 1). All tested echinocandins demonstrated a full EA between the Etest and CLSI methods, 100%, with the exception of anidulafungin, which exhibited an EA of 43.3% (Table 1). Figure 2 presents a representative clinical isolate classified as susceptible to anidulafungin by the CLSI method (MIC = 2 µg/mL) but resistant according to the Etest (MIC >32 µg/mL). Additionally, the figure includes an *fks* mutant isolate and quality controls for comparison.

### Reduced anidulafungin susceptibility unrelated to *fks*1 gene mutations

The sequencing results of the *fks*1 gene (HS1 and HS2 regions) in isolates that exhibited anidulafungin resistance by Etest were not correlated with any mutations in these regions (Table 3).

**TABLE 3:** Molecular analysis of *fks*1 mutations in *Candida parapsilosis* isolates with reduced susceptibility to anidulafungin (AND) by the Etest method

Isolate no. (*n* = 28)	Anidulafungin Etest MIC (mg/L)	Anidulafungin interpretation^*a*^	*fks*1 HS1^*b*^	*fks*1 HS2^*c*^
R1	≥32	Resistant	WT	WT
R3	24	Resistant	WT	WT
R4	≥32	Resistant	WT	WT
R5	12	Resistant	WT	WT
R6	≥32	Resistant	WT	WT
R8	≥32	Resistant	WT	WT
R9	≥32	Resistant	WT	WT
R10	≥32	Resistant	WT	WT
R11	≥32	Resistant	WT	WT
R13	24	Resistant	WT	WT
R14	12	Resistant	WT	WT
R16	16	Resistant	WT	WT
R17	≥32	Resistant	WT	WT
R18	≥32	Resistant	WT	WT
R20	≥32	Resistant	WT	WT
R22	≥32	Resistant	WT	WT
R23	32	Resistant	WT	WT
R24	≥32	Resistant	WT	WT
I2	16	Resistant	WT	WT
I4	12	Resistant	WT	WT
I7	8	Resistant	WT	WT
I13	8	Resistant	WT	WT
I19	12	Resistant	WT	WT
I21	≥32	Resistant	WT	WT
S1	8	Resistant	WT	WT
S9	≥32	Resistant	WT	WT
N1	32	Resistant	WT	WT
N2	≥32	Resistant	WT	WT

^*a*^ As there are no breakpoints established for the Etest method, the data were interpreted based on CLSI guidelines (25). The Etest results are presented in their actual, non-rounded form.

^*b*^ HS1, hot spot 1.

^*c*^ HS2, hot spot 2.

## DISCUSSION

Fluconazole has historically been the first-line therapy for managing candidal infections across various patient populations (8, 9). However, two major developments have necessitated a reevaluation of treatment strategies: the shift from *C. albicans*, generally susceptible to fluconazole, to non-*albicans Candida* species with varying azole susceptibilities (26, 27), and the rising incidence of azole resistance, particularly fluconazole resistance, among these non-*albicans* species (9, 28). With the introduction of echinocandins for managing invasive candidiasis, these agents have become the preferred first-line therapy of invasive candidiasis for adult patients (1, 29), showing efficacy comparable to fluconazole in randomized, comparative clinical trials (29). Anidulafungin is regarded as a leading drug for AFST against *C. parapsilosis* by the European Committee on Antimicrobial Susceptibility Testing guideline, and susceptibility to one echinocandin is often recommended to be extrapolated to other agents within the same class (30, 31).

Unlike relatively common azole resistance (9), echinocandin resistance in *C. parapsilosis* has historically been rare; however, recently identified cases of echinocandin-resistant strains pose an alarming threat (24, 32). *C. parapsilosis* naturally exhibits higher *in vitro* MICs to echinocandins compared to other *Candida* species, driven by a species-specific intrinsic substitution at amino acid position P660A in the *fks*1 protein, where alanine replaces proline in *C. parapsilosis*, leading to a reduced susceptibility phenotype to echinocandins (33, 34). The antifungal susceptibility profile of *Candida* species is a crucial factor in selecting the appropriate treatment, especially in invasive infections, prior antifungal exposure, suspected acquired drug resistance, or cases of unexpected treatment failure (1). For routine practice and optimal patient management, AFST techniques should be user-friendly, reproducible, low-cost, fast, and most importantly, accurate. Gradient diffusion strips, Etest, are commonly used in routine diagnostics, as they are less time-consuming and labor-intensive than broth microdilution (19).

Given that a part of our institute is routine diagnostics, we regularly participate in the annual quality control ring test, which provides external quality assessments for microbiological testing. During the quality control ring test, a discrepancy was noted between the anidulafungin AFST results obtained by the Etest (the method routinely used for AFST) and the broth microdilution methods (reference standard methods) for *C. parapsilosis* isolates. This led to differing MIC values and subsequent variation in categorization by each method, prompting the initiation of this study to investigate whether this variation is method-dependent. To our knowledge, few studies exclusively evaluated the agreement between standard broth microdilution methods and the commonly used Etest for *Candida* species (22, 35), and none have specifically focused on anidulafungin with an appropriate sample size of *C. parapsilosis*. Our AFST results indicated that anidulafungin was the sole echinocandin exhibiting method-dependent variability (Fig. 1 and Table 1), with a 43.3% EA between the Etest and CLSI methods. In contrast, the other echinocandins demonstrated 100% agreement between these methods (Table 1). None of the tested isolates were categorized as resistant to anidulafungin by the CLSI method, whereas 56.6% were classified as resistant by the Etest method (Table 1). To rule out Lot dependency of the anidulafungin Etest results, isolates showing inconsistencies between the Etest and CLSI methods were tested with a different Lot number, and the results showed no change in the final categorization, although some isolates exhibited variations within a 2-log dilution range (data not shown). The findings of the present study align with the limited available research (22, 35), suggesting that the Etest may not be reliable for testing anidulafungin against *C. parapsilosis* due to its low EA. However, multicenter studies and regular comparisons between centralized MIC determinations in reference laboratories and routine results from Etest methods could provide a more accurate assessment than a limited set of isolates.

Mutations in the hotspot regions of the *fks*1 gene are known to be associated with echinocandin resistance (15, 34) and can develop rapidly with short-term exposure to echinocandins (36). Therefore, in the present study, the sequences of the *fks* HS1 and HS2 regions were analyzed to determine if any mutations contributed to the elevated anidulafungin MIC values by Etest and the discrepancies in susceptibility results across different testing methods (Table 3). Given the absence of mutations, the observed discrepancies in anidulafungin susceptibility are more likely attributed to method-dependent factors. However, in general, to eliminate the possibility of mutations beyond the known hotspot regions, whole-genome sequencing or sequencing the entire *fks* gene in cases with wild-type HS regions would be warranted to identify potential novel mutations outside the well-known HS regions, as some studies have shown that not all mutations associated with echinocandin resistance are confined to the *fks* HS regions (16, 37, 38), and other potential mechanisms, aside from mutations, may also contribute to resistance (39).

To further evaluate this method-dependent discrepancy, we included previously published *fks1*^R658G^ mutant isolates in our study (31, 36) to determine whether the same pattern observed in clinical isolates also applies to fks mutants. Mutant strains had been previously classified as micafungin-resistant, anidulafungin-susceptible, and caspofungin-intermediate according to CLSI (31, 36). The Etest results of mutant strains reinforced our findings, as the anidulafungin Etest continued to show false resistance, whereas CLSI provided a more accurate classification. However, the results for the other echinocandins remained consistent between both methods, particularly for micafungin, where resistance was reliably detected by both Etest and CLSI.

The discrepancy between the Etest and broth microdilution methods is a recognized issue across various yeast and mold species with different antifungals, not limited to *Candida* and echinocandins (35, 40, 41). Although the exact cause of this variation is not fully understood and few studies address the potential factors, most of which focus on antibacterial and disk diffusion method (42, 43), it is likely due to several factors, primarily the natural differences between the agar-based Etest and the liquid-based CLSI method, which may influence the activity of certain antimycotics. One key factor may be the drug’s diffusion properties, which can vary between agar and broth media. The rate of diffusion of the agent through the agar depends on the drug’s diffusion and solubility properties, as well as the molecular weight of the compound. Larger molecules tend to diffuse more slowly than smaller ones (42, 43). Another variable could be the diffusion pattern. In agar-based methods, the agent diffuses in two dimensions, spreading horizontally across the surface, with the depth of the agar potentially influencing the zone of inhibition (42, 43). In contrast, diffusion in broth-based methods occurs in three dimensions, ensuring more uniform drug availability throughout the liquid medium, which may contribute to more consistent and reliable MIC determinations. As each antimycotic agent may exhibit a unique diffusion pattern, potentially leading to variations in the zone of inhibition and MIC readings, it is advisable to strengthen quality control measures or adjust testing methods, particularly when significant discrepancies are observed for a specific antifungal and species, rather than applying the same considerations to all agents within that class. In addition to these factors, the visual reading variability of the agar-and broth-based method might contribute further to the observed differences between the methods, influenced by individual interpretations (19), as well as differing endpoint criteria between the Etest and CLSI methods, growth reduction for Etest and for CLSI, since reading CLSI results is generally easier and less dependent on expertise than reading Etest results.

In conclusion, our study demonstrated that Etest results for anidulafungin against *C. parapsilosis* tend to overestimate resistance, and the susceptibility categorization obtained through this method cannot be reliably applied to other echinocandins due to method-dependent variability. In contrast, susceptibility results for other echinocandins against *C. parapsilosis* can be extended, as they show consistent outcomes across different methods (Table 1), in line with previous studies (22, 44). Considering the widespread use of the Etest method in diagnostic laboratories and the established role of anidulafungin as a leading echinocandin in AFST, whose susceptibility profiles may be extrapolated to other echinocandins, it is crucial to rigorously evaluate the reliability of this method in determining echinocandin susceptibility across specific species. Therefore, validating the accuracy of Etest in comparison to standard broth microdilution methods through quality control is essential, while considering the specific pattern of each agent within the same antifungal class and across different species, to ensure its reliable use in clinical settings. In addition, any necessary method adjustments or reading precautions should be advised in clinical settings, particularly for anidulafungin and *C. parapsilosis*.

## MATERIALS AND METHODS

### Fungal isolates

Sixty clinical isolates of *C. parapsilosis* were examined, including quality controls *C. parapsilosis* ATCC 22019, and *C. krusei* ATCC 6258. In addition to clinical isolates, three *C. parapsilosis* isolates harboring the *fks*1 mutation (R658G [A1972G]), which were previously characterized and classified as micafungin-resistant, anidulafungin-susceptible, and caspofungin-intermediate according to CLSI (23, 24), were also included in AFST. The isolates used in this study were selected from clinical samples collected over several years as part of routine diagnostics at the Institute of Hygiene and Medical Microbiology, Medical University of Innsbruck. These isolates were chosen to represent anidulafungin-resistant, intermediate, and susceptible categories, as determined by the Etest method used in routine diagnostic laboratory procedures. The specific taxonomical clade to which these isolates belong remains unidentified. As previously described (45), matrix-assisted laser desorption ionization time-of-flight mass spectrometry analysis was used to identify species level.

### Antifungal agents

Etest strips of anidulafungin (AND; 0.002–32 mg/L; BioMérieux, Vienna, Austria), micafungin (MYC; 0.002–32 mg/L; BioMérieux, Vienna, Austria), caspofungin (CAS; 0.002–32 mg/L; BioMérieux, Vienna, Austria), and rezafungin (RZF; 0.002–32 mg/L; Liofilchem, Roseto degli Abruzzi, TE, Italy) were utilized. Antifungal powders of anidulafungin (AND; 0.015–8 mg/L; Sigma-Aldrich, Vienna, Austria, SML2288) (solvent; dimethyl sulfoxide, Sigma-Aldrich, Vienna, Austria), micafungin (MYC; 0.015–8 mg/L; Sigma-Aldrich, Vienna, Austria, SML2268) (solvent; dimethyl sulfoxide, Sigma-Aldrich, Vienna, Austria), caspofungin (CAS; 0.015–8 mg/L; Sigma-Aldrich, Vienna, Austria, SML0425) (solvent; dimethyl sulfoxide, Sigma-Aldrich, Vienna, Austria), and rezafungin (RZF; 0.015–8 mg/L; MedChemExpress, Sollentuna, Sweden, HY-108009) (solvent; dimethyl sulfoxide, Sigma-Aldrich, Vienna, Austria) were used.

### Susceptibility testing and results interpretation

Before susceptibility testing, each isolate was sub-cultured from 10% glycerol frozen stocks (−80°C) on sabouraud dextrose agar (BioMérieux, Vienna, Austria, 1010158070) at 37°C for 24 h. *In vitro* susceptibilities to four echinocandins (anidulafungin, micafungin, caspofungin, and rezafungin) were determined in a single laboratory. Broth microdilution AFST was carried out according to the CLSI (25). As previously described (20), the Etest AFST was performed according to the manufacturer’s instructions, using ready-to-use RPMI 1640 agar (Axon-Lab, Tyrol, Austria, Lot No. 12048185). Briefly, inocula were prepared by transferring colonies from the 24-h-old cultures into 0.85% NaCl and adjusting the suspension to a McFarland standard turbidity equivalent of 0.5. The agar surface was inoculated with a swab dipped into the adjusted suspension and was left to get dry (about 15 min). The Etest strips were then applied to the agar surface, and the plates were incubated at 37°C. The same cell suspensions obtained from each isolate were used for the susceptibility testing of all four echinocandins.

MIC values of broth microdilution were interpreted according to CLSI M27-Ed4 (25). The Etest MIC results were evaluated at 24 h to detect the first visual point of significant growth inhibition (46). Both methods employed the CLSI breakpoints (M27M44S-Ed3) to classify the strains into three categories: susceptible, intermediate, and resistant (25). Isolates with MIC values of ≤2 µg/mL were classified as susceptible to all echinocandins, those with MIC values of 4 µg/mL were considered intermediate, and MIC values ≥8 µg/mL were categorized as resistant—except for rezafungin, where only the susceptible category (≤2 µg/mL) is defined, with no established intermediate or resistant classifications (47).

MIC_90_ represents the MIC values at which 90% of the tested isolates in a test population are inhibited. Etest MIC values that fell outside the microdilution range were rounded up to the nearest higher log2 dilution of broth microdilution. EA between Etest and broth microdilution results was considered when the MIC values obtained with the methods fell within ±2 dilutions of the twofold dilution scheme (48). However, if a difference of two dilutions resulted in a significant susceptibility reclassification (e.g., susceptible to resistant, and not intermediate), it was considered a non-consistent result. EA was then calculated as the percentage of isolates meeting these consistency criteria across all tested isolates, with EA values of ≥90% regarded as acceptable. Experiments were conducted in duplicate. When MIC values differed within ±2 dilutions of the twofold dilution scheme, the higher value was recorded; if they differed by more than two dilutions, the test was repeated.

### *fks1* gene sequencing

Isolates categorized as resistant based on actual, unrounded MIC values obtained by Etest, according to the CLSI clinical breakpoints, were selected for sequencing of the *fks1* gene. Genomic DNA was extracted from isolates grown for 18–24 h on SAB agar using the DNeasy UltraClean Microbial Kit (Qiagen, Germany). To identify mutations associated with echinocandin resistance, the hotspot regions (HS1 and HS2) of the *fks*1 gene from *C. parapsilosis* were sequenced as previously described (49), using the following previously published primers (49): HS1-F (5′-CAT ACR TTT ACT GCA AAC TTT GT-3′) and HS1-R (5′-GAT TTC CAT TTC GGT GGT-3′) for the HS1 region, and HS2-F (5′-TGC ATR TGA ACG AAG ATA TTT A-3′) and HS2-R (5′-GCA ACA AAR ACT TCA AAC AT-3′) for the HS2 region. The thermal cycling conditions were as follows, using PCR Kit (Q5 High-Fidelity, New England BioLabs, M0491L): initial denaturation at 95°C for 5 min, followed by 35 cycles of denaturation at 95°C for 30 s, annealing at 52°C for 30 s, and extension at 72°C for 30 s, with a final extension at 72°C for 8 min (49). The PCR products were visualized on a 1% agarose gel (Biozym, Germany, 840000) stained with GelRed nucleic acid gel stain (Biotium, USA, 23G0210). Successful amplification products were purified using the Exo-CIP Rapid PCR Cleanup Kit (New England BioLabs, USA, E1050S), as described by the manufacturer’s instructions. Sanger sequencing was performed for the aforementioned gene. After assembly and curation, sequence data were aligned against the WT *fks*1 sequence (33, 49), by BioNumerics 6.6 (Applied Maths NV, Belgium).

## Data Availability

Relevant supplementary material has been deposited in GenBank under accession nos. PQ741737 to PQ741792.

## References

[1] Pappas PG, Lionakis MS, Arendrup MC, Ostrosky-Zeichner L, Kullberg BJ. 2018. Invasive candidiasis. Nat Rev Dis Primers 4:1–20. doi:10.1038/nrdp.2018.26 29749387

[2] Cornely OA, Sprute R, Bassetti M, Chen SC-A, Groll AH, Kurzai O, Lass-Flörl C, Ostrosky-Zeichner L, Rautemaa-Richardson R, Revathi G, et al.. 2025. Global guideline for the diagnosis and management of candidiasis: an initiative of the ECMM in cooperation with ISHAM and ASM. Lancet Infect Dis:S1473-3099(24)00749-7. doi:10.1016/S1473-3099(24)00749-7 39956121

[3] Kullberg BJ, Arendrup MC. 2015. Invasive candidiasis. N Engl J Med 373:1445–1456. doi:10.1056/NEJMra1315399 26444731

[4] Arendrup MC. 2010. Epidemiology of invasive candidiasis. Curr Opin Crit Care 16:445–452. doi:10.1097/MCC.0b013e32833e84d2 20711075

[5] Pfaller MA, Andes DR, Diekema DJ, Horn DL, Reboli AC, Rotstein C, Franks B, Azie NE. 2014. Epidemiology and outcomes of invasive candidiasis due to non-albicans species of Candida in 2,496 Patients: data from the Prospective Antifungal Therapy (PATH) Registry 2004–2008. PLoS One 9:e101510. doi:10.1371/journal.pone.0101510 24991967 PMC4081561

[6] Cleveland AA, Farley MM, Harrison LH, Stein B, Hollick R, Lockhart SR, Magill SS, Derado G, Park BJ, Chiller TM. 2012. Changes in incidence and antifungal drug resistance in candidemia: results from population-based laboratory surveillance in Atlanta and Baltimore, 2008-2011. Clin Infect Dis 55:1352–1361. doi:10.1093/cid/cis697 22893576 PMC4698872

[7] Tóth R, Nosek J, Mora-Montes HM, Gabaldon T, Bliss JM, Nosanchuk JD, Turner SA, Butler G, Vágvölgyi C, Gácser A. 2019. Candida parapsilosis: from genes to the bedside. Clin Microbiol Rev 32:e00111-18. doi:10.1128/CMR.00111-18 30814115 PMC6431126

[8] Govrins M, Lass-Flörl C. 2024. Candida parapsilosis complex in the clinical setting. Nat Rev Microbiol 22:46–59. doi:10.1038/s41579-023-00961-8 37674021

[9] Daneshnia F, de Almeida Júnior JN, Ilkit M, Lombardi L, Perry AM, Gao M, Nobile CJ, Egger M, Perlin DS, Zhai B, Hohl TM, Gabaldón T, Colombo AL, Hoenigl M, Arastehfar A. 2023. Worldwide emergence of fluconazole-resistant Candida parapsilosis: current framework and future research roadmap. Lancet Microbe 4:e470–e480. doi:10.1016/S2666-5247(23)00067-8 37121240 PMC10634418

[10] Branco J, Miranda IM, Rodrigues AG. 2023. Candida parapsilosis virulence and antifungal resistance mechanisms: a comprehensive review of key determinants. J Fungi (Basel) 9:80. doi:10.3390/jof9010080 36675901 PMC9862255

[11] Pappas PG, Kauffman CA, Andes DR, Clancy CJ, Marr KA, Ostrosky-Zeichner L, Reboli AC, Schuster MG, Vazquez JA, Walsh TJ, Zaoutis TE, Sobel JD. 2016. Clinical practice guideline for the management of candidiasis: 2016 update by the infectious diseases society of America. Clin Infect Dis 62:e1–e50. doi:10.1093/cid/civ933 26679628 PMC4725385

[12] Pfaller MA, Boyken L, Hollis RJ, Kroeger J, Messer SA, Tendolkar S, Diekema DJ. 2008. In vitro susceptibility of invasive isolates of Candida spp. to anidulafungin, caspofungin, and micafungin: six years of global surveillance. J Clin Microbiol 46:150–156. doi:10.1128/JCM.01901-07 18032613 PMC2224271

[13] Kontoyiannis DP, Bassetti M, Nucci M, Capparella MR, Yan JL, Aram J, Hogan PA. 2017. Anidulafungin for the treatment of candidaemia caused by Candida parapsilosis: analysis of pooled data from six prospective clinical studies. Mycoses 60:663–667. doi:10.1111/myc.12641 28597967

[14] Szymański M, Chmielewska S, Czyżewska U, Malinowska M, Tylicki A. 2022. Echinocandins - structure, mechanism of action and use in antifungal therapy. J Enzyme Inhib Med Chem 37:876–894. doi:10.1080/14756366.2022.2050224 35296203 PMC8933026

[15] Perlin DS. 2015. Echinocandin resistance in Candida. Clin Infect Dis 61:S612–S617. doi:10.1093/cid/civ791 26567278 PMC4643482

[16] Daneshnia F, Arastehfar A, Lombardi L, Binder U, Scheler J, Vahedi Shahandashti R, Hagen F, Lass-Flörl C, Mansour MK, Butler G, Perlin DS. 2023. Candida parapsilosis isolates carrying mutations outside FKS1 hotspot regions confer high echinocandin tolerance and facilitate the development of echinocandin resistance. Int J Antimicrob Agents 62:106831. doi:10.1016/j.ijantimicag.2023.106831 37121442

[17] Zhai B, Liao C, Jaggavarapu S, Tang Y, Rolling T, Ning Y, Sun T, Bergin SA, Gjonbalaj M, Miranda E, Babady NE, Bader O, Taur Y, Butler G, Zhang L, Xavier JB, Weiss DS, Hohl TM. 2024. Antifungal heteroresistance causes prophylaxis failure and facilitates breakthrough Candida parapsilosis infections. Nat Med 30:3163–3172. doi:10.1038/s41591-024-03183-4 39095599 PMC11840754

[18] Sipsas NV, Lewis RE, Tarrand J, Hachem R, Rolston KV, Raad II, Kontoyiannis DP. 2009. Candidemia in patients with hematologic malignancies in the era of new antifungal agents (2001‐2007). Cancer 115:4745–4752. doi:10.1002/cncr.24507 19634156

[19] Berkow EL, Lockhart SR, Ostrosky-Zeichner L. 2020. Antifungal susceptibility testing: current approaches. Clin Microbiol Rev 33:e00069-19. doi:10.1128/CMR.00069-19 32349998 PMC7194854

[20] Vahedi-Shahandashti R, Hahn L, Houbraken J, Lass-Flörl C. 2023. Aspergillus section terrei and antifungals: from broth to agar-based susceptibility testing methods. J Fungi (Basel) 9:306. doi:10.3390/jof9030306 36983474 PMC10056208

[21] Axner-Elings M, Botero-Kleiven S, Jensen RH, Arendrup MC. 2011. Echinocandin susceptibility testing of Candida isolates collected during a 1-year period in Sweden. J Clin Microbiol 49:2516–2521. doi:10.1128/JCM.00201-11 21543574 PMC3147820

[22] Espinel-Ingroff A, Canton E, Peman J, Martín-Mazuelo E. 2010. Comparison of anidulafungin MICs determined by the clinical and laboratory standards institute broth microdilution method (M27-A3 document) and Etest for Candida species isolates. Antimicrob Agents Chemother 54:1347–1350. doi:10.1128/AAC.01324-09 20028814 PMC2825960

[23] Daneshnia F, Hilmioğlu-Polat S, Ilkit M, Fuentes D, Lombardi L, Binder U, Scheler J, Hagen F, Mansour MK, Butler G, Lass-Flörl C, Gabaldon T, Arastehfar A. 2023. Whole-genome sequencing confirms a persistent candidaemia clonal outbreak due to multidrug-resistant Candida parapsilosis. J Antimicrob Chemother 78:1488–1494. doi:10.1093/jac/dkad112 37100456 PMC10232251

[24] Arastehfar A, Daneshnia F, Hilmioglu-Polat S, Ilkit M, Yasar M, Polat F, Metin DY, Dokumcu ÜZ, Pan W, Hagen F, Boekhout T, Perlin DS, Lass-Flörl C. 2021. Genetically related micafungin-resistant Candida parapsilosis blood isolates harbouring novel mutation R658G in hotspot 1 of Fks1p: a new challenge? J Antimicrob Chemother 76:418–422. doi:10.1093/jac/dkaa419 33175162

[25] CLSI. 2017. CLSI standard M27. Reference method for broth dilution antifungal susceptibility testing of yeasts. 4th ed. Clinical and Laboratory Standards Institute, Wayne, PA.

[26] Seyoum E, Bitew A, Mihret A. 2020. Distribution of Candida albicans and non-albicans Candida species isolated in different clinical samples and their in vitro antifungal suscetibity profile in Ethiopia. BMC Infect Dis 20:231. doi:10.1186/s12879-020-4883-5 32188422 PMC7081544

[27] Whaley SG, Berkow EL, Rybak JM, Nishimoto AT, Barker KS, Rogers PD. 2017. Azole antifungal resistance in Candida albicans and emerging non-albicans Candida species. Front Microbiol 7:2173. doi:10.3389/fmicb.2016.02173 28127295 PMC5226953

[28] Berkow EL, Lockhart SR. 2017. Fluconazole resistance in Candida species: a current perspective. Infect Drug Resist 10:237–245. doi:10.2147/IDR.S118892 28814889 PMC5546770

[29] Reboli AC, Rotstein C, Pappas PG, Chapman SW, Kett DH, Kumar D, Betts R, Wible M, Goldstein BP, Schranz J, Krause DS, Walsh TJ, Anidulafungin Study Group. 2007. Anidulafungin versus fluconazole for invasive candidiasis. N Engl J Med 356:2472–2482. doi:10.1056/NEJMoa066906 17568028

[30] European Committee on Antimicrobial Susceptibility Testing. 2020. Anidulafungin: rationale for the clinical breakpoints, version 3.0, 2020. Available from: http://www.eucast.org

[31] Arendrup MC, Friberg N, Mares M, Kahlmeter G, Meletiadis J, Guinea J, Arendrup MC, Meletiadis J, Guinea J, Friberg N, et al.. 2020. How to interpret MICs of antifungal compounds according to the revised clinical breakpoints v. 10.0 European committee on antimicrobial susceptibility testing (EUCAST). Clin Microbiol Infect 26:1464–1472. doi:10.1016/j.cmi.2020.06.007 32562861

[32] Ning Y, Xiao M, Perlin DS, Zhao Y, Lu M, Li Y, Luo Z, Dai R, Li S, Xu J, Liu L, He H, Liu Y, Li F, Guo Y, Chen Z, Xu Y, Sun T, Zhang L. 2023. Decreased echinocandin susceptibility in Candida parapsilosis causing candidemia and emergence of a pan-echinocandin resistant case in China. Emerg Microbes Infect 12:2153086. doi:10.1080/22221751.2022.2153086 36440795 PMC9793909

[33] Garcia-Effron G, Katiyar SK, Park S, Edlind TD, Perlin DS. 2008. A naturally occurring proline-to-alanine amino acid change in Fks1p in Candida parapsilosis, Candida orthopsilosis, and Candida metapsilosis accounts for reduced echinocandin susceptibility. Antimicrob Agents Chemother 52:2305–2312. doi:10.1128/AAC.00262-08 18443110 PMC2443908

[34] Cowen LE, Sanglard D, Howard SJ, Rogers PD, Perlin DS. 2015. Mechanisms of antifungal drug resistance. Cold Spring Harb Perspect Med 5:a019752. doi:10.1101/cshperspect.a019752 PMC4484955 25384768

[35] Arendrup MC, Garcia-Effron G, Lass-Flörl C, Lopez AG, Rodriguez-Tudela J-L, Cuenca-Estrella M, Perlin DS. 2010. Echinocandin susceptibility testing of Candida species: comparison of EUCAST EDef 7.1, CLSI M27-A3, Etest, disk diffusion, and agar dilution methods with RPMI and isosensitest media. Antimicrob Agents Chemother 54:426–439. doi:10.1128/AAC.01256-09 19884370 PMC2798528

[36] Khalifa HO, Watanabe A, Kamei K. 2024. Genetic mutations in FKS1 gene associated with acquired echinocandin resistance in Candida parapsilosis complex. Mycopathologia 189:40. doi:10.1007/s11046-024-00847-0 38704798

[37] Rybak JM, Dickens CM, Parker JE, Caudle KE, Manigaba K, Whaley SG, Nishimoto AT, Luna-Tapia A, Roy S, Zhang Q, Barker KS, Palmer GE, Sutter TR, Homayouni R, Wiederhold NP, Kelly SL, Rogers PD. 2017. Loss of C-5 sterol desaturase activity results in increased resistance to azole and echinocandin antifungals in a clinical isolate of Candida parapsilosis. Antimicrob Agents Chemother 61:e00651-17. doi:10.1128/AAC.00651-17 28630186 PMC5571332

[38] Martí-Carrizosa M, Sánchez-Reus F, March F, Cantón E, Coll P. 2015. Implication of Candida parapsilosis FKS1 and FKS2 mutations in reduced echinocandin susceptibility. Antimicrob Agents Chemother 59:3570–3573. doi:10.1128/AAC.04922-14 25779577 PMC4432215

[39] Sun L-L, Li H, Yan T-H, Cao Y-B, Jiang Y-Y, Yang F. 2023. Aneuploidy enables cross-tolerance to unrelated antifungal drugs in Candida parapsilosis. Front Microbiol 14:1137083. doi:10.3389/fmicb.2023.1137083 37113223 PMC10126355

[40] Serrano MC, Morilla D, Valverde A, Chávez M, Espinel-Ingroff A, Claro R, Ramírez M, Mazuelos EM. 2003. Comparison of Etest with modified broth microdilution method for testing susceptibility of Aspergillus spp. to voriconazole. J Clin Microbiol 41:5270–5272. doi:10.1128/JCM.41.11.5270-5272.2003 14605181 PMC262461

[41] Dannaoui E, Paugam A, Develoux M, Chochillon C, Matheron J, Datry A, Bouges-Michel C, Bonnal C, Dromer F, Bretagne S. 2010. Comparison of antifungal MICs for yeasts obtained using the EUCAST method in a reference laboratory and the Etest in nine different hospital laboratories. Clin Microbiol Infect 16:863–869. doi:10.1111/j.1469-0691.2009.02997.x 19778296

[42] Jorgensen JH, Turnidge JD. 2015. Susceptibility test methods: dilution and disk diffusion methods, p 1253–1273. In Manual of clinical microbiology. John Wiley & Sons, Ltd.

[43] American Society for Microbiology. 2025. Kirby-Bauer disk diffusion susceptibility test protocol. ASM.Org. Available from: https://asm.org:443/Protocols/Kirby-Bauer-Disk-Diffusion-Susceptibility-Test-Pro

[44] Marcos-Zambrano LJ, Escribano P, Rueda C, Zaragoza Ó, Bouza E, Guinea J. 2013. Comparison between the EUCAST procedure and the Etest for determination of the susceptibility of Candida species isolates to micafungin. Antimicrob Agents Chemother 57:5767–5770. doi:10.1128/AAC.01032-13 23979756 PMC3811256

[45] Samantaray S, Singh R. 2022. Evaluation of MALDI-TOF MS for identification of species in the Candida parapsilosis complex from candidiasis cases. J Appl Lab Med 7:889–900. doi:10.1093/jalm/jfac005 35348720

[46] Dannaoui E, Espinel-Ingroff A. 2019. Antifungal susceptibly testing by concentration gradient strip Etest method for fungal isolates: a review. J Fungi (Basel) 5:108. doi:10.3390/jof5040108 31766762 PMC6958406

[47] CLSI. 2021. Subcommittee (SC) on antifungal susceptibility tests. June 2021 meeting minutes. https://clsi.org/media/gvuivvig/2021_summer_afsc_agenda_summary_minutes.pdf.

[48] Espinel-Ingroff A. 2001. Comparison of the E-test with the NCCLS M38-P method for antifungal susceptibility testing of common and emerging pathogenic filamentous fungi. J Clin Microbiol 39:1360–1367. doi:10.1128/JCM.39.4.1360-1367.2001 11283057 PMC87940

[49] Arastehfar A, Daneshnia F, Najafzadeh MJ, Hagen F, Mahmoudi S, Salehi M, Zarrinfar H, Namvar Z, Zareshahrabadi Z, Khodavaisy S, Zomorodian K, Pan W, Theelen B, Kostrzewa M, Boekhout T, Lass-Flörl C. 2020. Evaluation of molecular epidemiology, clinical characteristics, antifungal susceptibility profiles, and molecular mechanisms of antifungal resistance of Iranian Candida parapsilosis species complex blood isolates. Front Cell Infect Microbiol 10:206. doi:10.3389/fcimb.2020.00206 32509592 PMC7253641

